# Dramatic Clinical Response of Relapsed Metastatic Extramammary Paget's Disease to Trastuzumab Monotherapy

**DOI:** 10.1155/2012/401362

**Published:** 2012-03-27

**Authors:** S. Wakabayashi, Y. Togawa, K. Yoneyama, K. Suehiro, N. Kambe, H. Matsue

**Affiliations:** Department of Dermatology, Chiba University Graduate School of Medicine, 1-8-1 Inohana, Chuo-ku, Chiba 260-8670, Japan

## Abstract

We report the first case of 68-year-old Japanese woman with metastatic HER2-positive extramammary Paget's disease that showed the validity of trastuzumab monotherapy. We administered trastuzumab at a loading dose of 8 mg/kg i.v., followed by a 6 mg/kg maintenance dose every three weeks according to a protocol for HER2-positive metastatic breast cancers and a near-complete response was achieved after the tenth infusion. The patient experienced a moderate headache and flushing during the first infusion, but had no advanced effects during subsequent infusions with ibuprofen and d-chlorpheniramine maleate. Given the dramatic response, the patient has had 17 infusions of trastuzumab with no disease progression. Thus, trastuzumab has few side effects and is well tolerated for elderly patients. It may become a new choice of the adjubant therapy of this disease.

## 1. Introduction

Extramammary Paget's disease (EMPD) is considered to be a malignant cutaneous tumor of apocrine gland origin. Advanced EMPD frequently metastasizes to the lymph nodes and abdominal viscera. EMPD often recurs years after radical surgery, with a poor prognosis. Though adjuvant therapy is employed, there is little published data regarding chemotherapy for advanced EMPD. 

## 2. Case Presentation

A 68-year-old Japanese woman with EMPD of the right labia major extending to the urethra, vaginal wall, and the uterine cervix was initially treated with a wide local excision and radical hysterectomy in January 2002. Histological examination confirmed EMPD with negative margins and lymph nodes. She did not receive adjuvant therapy but remained disease-free for five years until computer tomography (CT) revealed a 24 × 17 mm tumor in the right pelvic floor. The recurrent tumor was resected, and the patient remained disease free for the next three years. 

**Figure 1:**
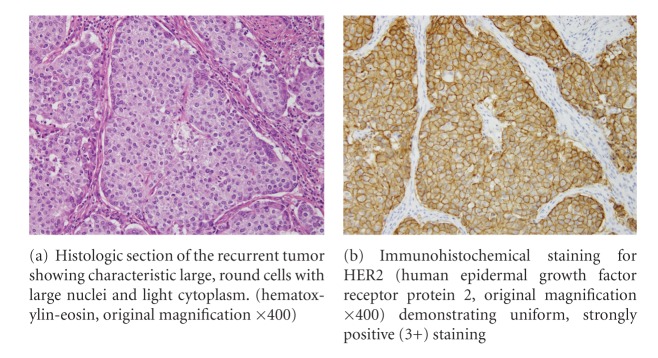
Histologic and immunohistochemical findings of the recurrent tumor.

 In March 2010, a CT scan demonstrated multiple enlarged pelvic, para-aortic, and mesenteric lymph nodes, which were suspicious for metastasis, as well as nodules in the lingulare superius of the left lung and segment VIII in the liver. Immunohistochemical analysis of a tumor specimen taken from the second surgery demonstrated diffuse positive staining for gross cystic disease fluid protein 15 (GCDFP-15) as an antibody specific with EMPD and also strongly positive (3+) staining for human epidermal growth factor receptor protein 2 (HER2) (Figure 1). We administered trastuzumab as monotherapy because she was of advanced age, at a loading dose of 8 mg/kg i.v., followed by a 6 mg/kg maintenance dose every three weeks according to a protocol for HER2-positive metastatic breast cancers [1]. The patient experienced a moderate headache and flushing during the first infusion, but had no adverse effects during subsequent infusions as she was pretreated with ibuprofen and d-chlorpheniramine maleate.

**Figure 2:**
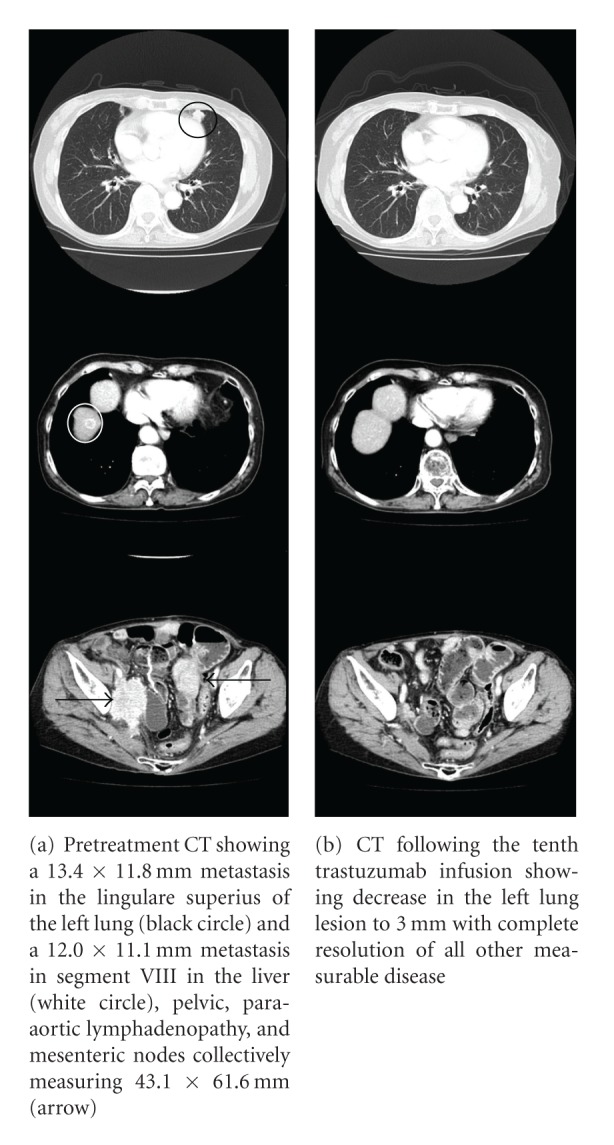
CT image of the metastatic lesion.

 In August 2010, the third trastuzumab infusion achieved a partial response as evidenced by decreases in the measurements of the pelvic, para-aortic, and mesenteric lymph nodes by CT scan. The metastases at the lingulare superius of the left lung and liver also decreased in size significantly. No new metastasis was detected. Following the tenth infusion, a near-complete response was achieved with the only residual disease being a 3 mm, scar-like shadow in the left lung (Figure 2). Given the dramatic response, maintenance trastuzumab infusions have been continued, and the patient has had 17 infusions thus far with no disease progression.

## 3. Discussion

Herceptin demonstrates antitumor effects after combination specifically to HER2 receptor by antibody-dependent cell-mediated cytotoxicity of NK cell and the monocyte. In randomized trial, trastuzumab plus docetaxel therapy is significantly superior to trastuzumab monotherapy followed by trastuzumab plus docetaxel therapy in patients with HER2-positive metastatic breast cancer, especially in terms of overall survival [2]. Therefore, many case reports document efficacy of trastuzumab against EMPD, when used in combination with docetaxel [3, 4]. In this case, trastuzumab monotherapy achieved a dramatic response with eventual disease stabilization. We chose trastuzumab as the tumor cells uniformly stained positive for HER2 protein. Additionally, trastuzumab has few side effects and is well tolerated for elderly patients.

 The efficacy of the present trastuzumab regimen has already been established for HER2-positive breast cancers; however, it is not widely used for EMPD despite 20–60% being positive for HER2 [5, 6]. There are no reports of trastuzumab monotherapy for EMPD. We report the first case that showed the validity of trastuzumab monotherapy for advanced age patient of metastatic EMPD. Moreover, this therapy may become a new choice of the adjuvant therapy of this disease.

## References

[1] Slamon DJ, Leyland-Jones B, Shak S (2001). Use of chemotherapy plus a monoclonal antibody against her2 for metastatic breast cancer that overexpresses HER2. *The New England Journal of Medicine*.

[2] Inoue K, Nakagami K, Mizutani M (2010). Randomized phase III trial of trastuzumab monotherapy followed by trastuzumab plus docetaxel versus trastuzumab plus docetaxel as first-line therapy in patients with HER2-positive metastatic breast cancer: the JO17360 Trial Group. *Breast Cancer Research and Treatment*.

[3] Takahagi S, Noda H, Kamegashira A (2009). Metastatic extramammary Paget’s disease treated with paclitaxel and trastuzumab combination chemotherapy. *The Journal of Dermatology*.

[4] Yagishita Y, Maekawa T (2010). Weekly-docetaxel combination chemotherapy induced CR in advanced extramammary Paget’s disease. *Skin Cancer*.

[5] Ogawa T, Nagashima Y, Wada H (2005). Extramammary Paget’s disease: analysis of growth signal pathway from the human epidermal growth factor receptor 2 protein. *Human Pathology*.

[6] Takata M, Fujimoto A, Aoki H, Hatta N, Ooi A, Takehara K (1999). erbB-2 overexpression but no activation of *β*-catenin gene in extramammary Paget’s disease. *Journal of Investigative Dermatology*.

